# Comparing the Thermal and Electrochemical Stabilities of Two Structurally Similar Ionic Liquids

**DOI:** 10.3390/molecules25102388

**Published:** 2020-05-21

**Authors:** Faiz Ullah Shah, Inayat Ali Khan, Patrik Johansson

**Affiliations:** 1 Chemistry of Interfaces, Luleå University of Technology, SE-971 87 Luleå, Sweden; inayat.khan@ltu.se; 2 Department of Physics, Chalmers University of Technology, SE-412 96 Gothenburg, Sweden

**Keywords:** ionic liquids, thermo-gravimetric analysis, ionic conductivity, electrochemical stability

## Abstract

Here we focus on the thermal and variable temperature electrochemical stabilities of two ionic liquids (ILs) having a common tributyloctyl phosphonium cation [P_4,4,4,8_]^+^ and two different orthoborate anions: bis(mandelato)borate [BMB]^−^ and bis(salicylato)borate [BScB]^−^. The thermo-gravimetric analysis data suggest that [P_4,4,4,8_][BScB] is thermally more stable than [P_4,4,4,8_][BMB] in both nitrogen atmosphere and air, while the impedance spectroscopy reveals that [P_4,4,4,8_][BScB] has higher ionic conductivity than [P_4,4,4,8_][BMB] over the whole studied temperature range. In contrast, the electrochemical studies confirm that [P_4,4,4,8_][BMB] is more stable and exhibits a wider electrochemical stability window (ESW) on a glassy carbon electrode surface as compared to [P_4,4,4,8_][BScB]. A continuous decrease in the ESWs of both ILs is observed as a function of operation temperature.

## 1. Introduction

Ionic liquids (ILs) are salts comprising cations and anions that form three-dimensional mobile and dynamic networks in the bulk phase. ILs are low melting salts (also known as molten salts) due to the large sizes, the nature of the molecular structures, and the high degree of asymmetry of the cations and anions. These structural features lead to weak Coulombic attractions between the ions of ILs as compared to inorganic salts [1,2,3,4]. ILs possess remarkable properties when compared with molecular liquids, making them attractive alternatives for a wide range of applications. Some of the properties of ILs include high polarity, high thermal stability (up to 450 °C), non-volatility, high ionic conductivity, wide liquid temperature range, and structural designability [5,6]. The outstanding properties of ILs enable them to be promising solvents in organic synthesis [7], catalysis [8,9], lubrication [10], pharmaceuticals [11], electrochemistry [12], energy storage devices [13,14], liquid-liquid extractions [15], extraction of metals [16,17], and separation of gases [18,19].

Thermal stability of ILs is very crucial in almost all of the above-mentioned applications. A too low thermal stability might restrict the usage in various applications. However, the optimum thermal stability depends on the operation conditions of a particular application. Thermal stability of ILs is most often assessed by using thermo-gravimetric analysis (TGA) [20]. The thermal stability recorded of any IL directly depends on the purity, the atmosphere used in the TGA (usually air or an inert gas), the gas flow rate, the heating rate, and the sample mass [21]. Generally, TGA obtained thermal stabilities of ILs are defined by the *T*_onset_, which is calculated from the intersection of the baseline weight and the tangent of the weight loss as a function of temperature [22]. It is known that the nature of both cation and anion plays an important role in governing the thermal decomposition of ILs. Tsunashima et al. found that benzyl-substituted phosphonium ILs have much higher thermal stabilities than the analogous benzyl-substituted ammonium ILs having a common bis(trifluoromethylsulfonyl)imide (TFSI) anion [23]. In another study, comparing different ILs with a common phosphonium cation [P_6,6,6,14_]^+^ and different anions, the thermal stability was found to follow the order TFSI > [FAP]^−^ > [N(CN)_2_]^−^ > Cl^−^ > [MeSO_4_]^−^ [24].

Like thermal stability, electrochemical stability of ILs is an extremely important property for electrochemical applications. The electrochemical stability window (ESW) is the potential difference between the oxidation and reduction reactions and mainly depends on the nature of both cation and anion as well as the electrode material(s) used. ILs as solvents and/or electrolytes have shown numerous advantages over molecular liquids in electrochemical devices. The ESWs of ILs have been shown to exceed 6.5 V, which is significantly larger than the ESWs of conventional organic solvents used for electrolytes; for acetonitrile the ESW is 5.0 V, for dichloromethane 3.5 V, and for dimethylsulfoxide 4.4 V [25]. It is also important to know the resulting decomposition products. When a potential wider than the ESW of the IL [C_4_mpyr]TFSI was applied, this IL decomposed into methylpyrrolidine, octanes, octenes, 2-butanol, dibutylmethylamine and butylpyrrolidine [26].

**Figure 1:**
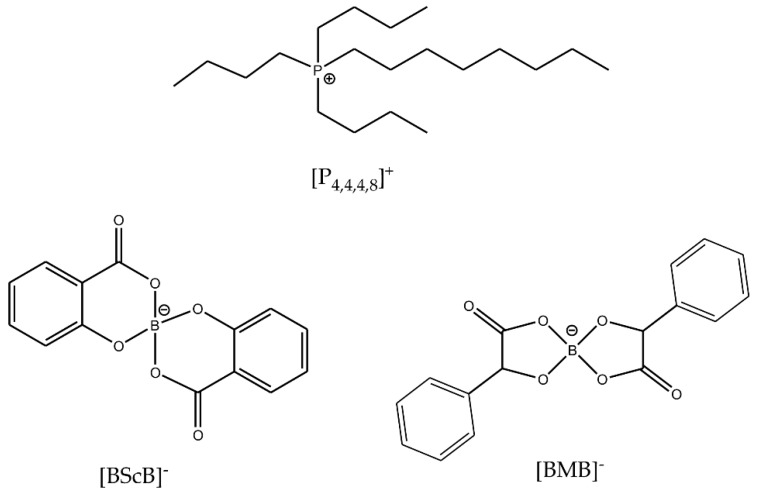
Chemical structures and abbreviations of the IL ions.

Thermal and electrochemical stabilities of ILs can be improved by an appropriate selection and combination of the cations and anions. In this work, we have chosen to study two ILs with a common [P_4,4,4,8_]^+^ cation and two different orthoborate anions: [BMB]^−^ and [BScB]^−^ (Figure 1). The choice of these two particular ILs is based on the similar chemical structures of the anions, but very different reactivity with inorganic oxide surfaces [27]. Both anions have an orthoborate (BO_4_) central core, two phenyl rings and two carbonyl groups. The only difference is the presence of two >CH- chemical moieties in the structure of the [BMB]^−^ anion. In our previous studies, the [BMB]^−^ anion in [P_6,6,6,14_][BMB] IL exhibited significant changes in the structure at the surfaces of inorganic oxides such as γ-Al_2_O_3_, MgO and SiO_2_ as revealed by multinuclear (^11^B, ^31^P and ^29^Si) solid-state MAS NMR, IR and Raman spectroscopies while the [BScB]^−^ anion in the [P_6,6,6,14_][BScB] IL remained unchanged, even after heating at 110 °C for 15 h [27].

Sustainable chemical products in general refer to the concept of ecotoxicological risk profiles, which comparatively assess five risk factors: release, spatiotemporal range, bioaccumulation, biological activity and uncertainty, and ILs often provide low risks for each of these indicators [28]. The ILs investigated here are halogen-free, hydrophobic and hydrolytically stable, which reduces the risks of corrosion, lowers the toxicity towards aquatic life and facilitates recyclability from aqueous systems, pointing to overall ecological and economical sustainability. Here we focus on the [P_4,4,4,8_][BMB] and [P_4,4,4,8_][BScB] ILs and investigate their thermal stability both in nitrogen atmosphere and in air, and their electrochemical stability on the surface of a glassy carbon (GC) electrode as a function of temperature.

## 2. Results and Discussion

### 2.1. Thermal Stability

**Figure 2:**
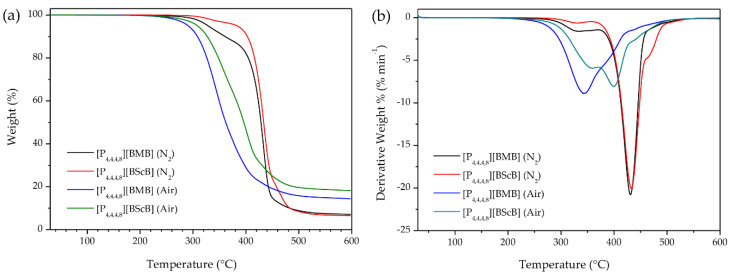
(**a**) TGA thermograms and (**b**) DTG curves of ILs under nitrogen atmosphere and in air at a heating rate of 10 °C min^−1^.

Since the applied atmosphere has a significant role in determining the thermal stability of ILs, we measured TGA both in dry nitrogen atmosphere and in air. Assessing the thermal stability of ILs under air is very important because in many practical applications the samples are in direct contact with air. Figure 2a shows the TGA curves of the [P_4,4,4,8_][BMB] and [P_4,4,4,8_][BScB] ILs under nitrogen and air using identical experimental conditions. Both ILs show major weight loss in a single step, indicating that both the cation and the anion decompose within a narrow temperature range. The TGA data show that [P_4,4,4,8_][BScB] is thermally more stable (*T*_onset_ are 412 °C in N_2_ and 322 °C in air) than [P_4,4,4,8_][BMB] (*T*_onset_ are 403 °C in N_2_ and 305 °C in air). Thus, both ILs decompose at much lower temperatures in air, but despite these lower decomposition temperatures, 14.4 wt.% and 18.2 wt.% of [P_4,4,4,8_][BMB] and [P_4,4,4,8_][BScB], respectively, remained after heating to 600 °C. On the other hand, only 7.1 wt.% and 6.5 wt.% of the decomposition products from [P_4,4,4,8_][BMB] and [P_4,4,4,8_][BScB], respectively, remained after heating to 600 °C in nitrogen atmosphere. This is in agreement with a previously published report on the thermal stability of phosphonium-based ILs [29] stating that the decomposition of phosphonium-based ILs under air might lead to the formation of tertiary phosphines, through a reversed Menshutkin reaction, which can be further oxidized to form tertiary phosphine oxides, which are thermally very stable species [30]. The lower thermal stability of these ILs in air might be due to oxidation of the [P_4,4,4,8_]^+^ cation followed by an accelerated decomposition of the [BMB]^−^ and [BScB]^−^ anions.

The derivative thermogravimetric (DTG) curves, i.e., weight loss per time-unit and temperature, showed that the rate of weight loss for both [P_4,4,4,8_][BMB] (21 wt.% min^−1^) and [P_4,4,4,8_][BScB] (20 wt.% min^−1^) under N_2_ is maximal at ca. 430 °C (with shoulders on both sides) (Figure 2b). However, this changes when exposed to air; the highest rates of weight loss are much lower: 9 wt.% min^−1^ at 344 °C for [P_4,4,4,8_][BMB] and 8 wt.% min^−1^ at 402 °C for [P_4,4,4,8_][BScB], again with shoulders on one side ([P_4,4,4,8_][BMB]) and both sides ([P_4,4,4,8_][BScB]). The lower rates of weight loss might be due to a high thermal stability of the tertiary phosphine oxides formed.

The thermal stabilities of these ILs are better and/or comparable with other commonly studied ILs based on imidazolium, ammonium and phosphonium cations combined with conventional anions such as halides, BF_4_, PF_6_, etc. Ngo et al. studied a range of imidazolium-based ILs with halogenated anions and most decomposed below 400 °C, with the exception of ILs with the TFSI anion [31]. For ten different phosphonium and ammonium cation-based ILs with a common dicyanamide anion, the phosphonium-based were found to be relatively more stable (*T*_dec_ < 400 °C) than the ammonium-based (*T*_dec_ < 300 °C) [32].

### 2.2. Ionic Conductivity

**Figure 3:**
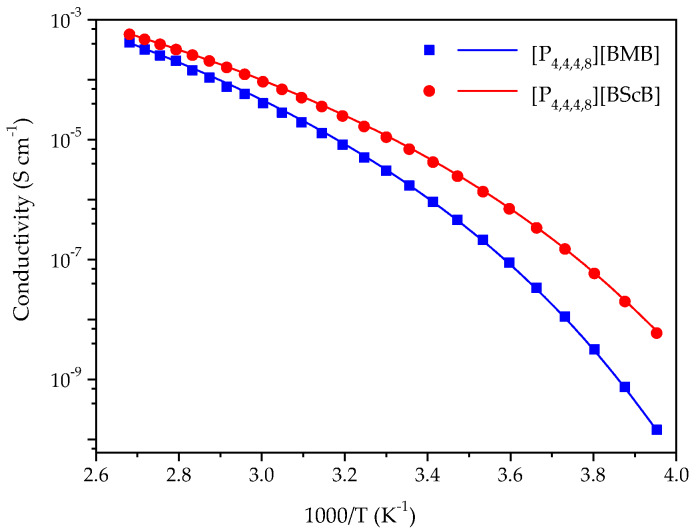
Temperature dependence of the ionic conductivities for [P_4,4,4,8_][BMB] and [P_4,4,4,8_][BScB] and the best fit of data using the VFT equation as indicated by the solid lines.

Ionic conductivity of ILs is generally controlled by the size and molecular weight of cation and anion, density and viscosity, and the interactions between the ions-the latter is one the main reasons for ion-pairing, and thereby reduced ionic conductivity [33,34,35]. A comparison of the ionic conductivities of the [P_4,4,4,8_][BMB] and [P_4,4,4,8_][BScB] ILs in the range from −20 °C to 100 °C is shown in Figure 3, wherein [P_4,4,4,8_][BMB] has lower ionic conductivity than [P_4,4,4,8_][BScB] throughout the whole temperature range. This was expected as [P_4,4,4,8_][BMB] has a higher viscosity than [P_4,4,4,8_][BScB] [36]. It is well known that viscous ILs with bulky and asymmetric ions display lower ionic conductivities due to hindered mobility of ions [37]. The higher ionic conductivity of [P_4,4,4,8_][BScB] can also be justified by the relatively lower molecular weight of the [BScB]^−^ anion (283.0 g mol^−1^) as compared to the [BMB]^−^ anion (311.0 g mol^−1^) and its more compact chemical structure.

The ionic conductivity data were analyzed further by fitting to the Vogel-Fulcher-Tammann (VFT) equation, which is commonly used to describe the ionic conductivity of ILs [38]:

(1) $$\sigma =\sigma_{0}\exp\left(\frac{-B}{\left(T-T_{0}\right)}\right)$$

where σ_0_, *B*, and *T*_0_ are fitting parameters: a pre-exponential factor, a factor related to the activation and the ideal glass transition temperature, respectively. The activation energy for ionic conductivity (Eσ) is related to B as Eσ = *B* × *R*. The fitting procedure was carried out over the full temperature range in two steps. In the first step, we plot ln(σ) vs. 1/(*T−T*_0_) and selected *T*_0_ to have a linear dependence. In the second step, we fit the dependence by a linear regression to obtain the fitting parameters (σ_0_, *B*). The VFT parameters (Table 1) show that the *E*σ of [P_4,4,4,8_][BMB] is slightly higher than for [P_4,4,4,8_][BScB], which was expected, as the ionic conductivity of the latter is higher. This reveals that relatively lower thermal energy is required for ion mobility in the case of [P_4,4,4,8_][BScB].

### 2.3. Electrochemical Stability

**Figure 4:**
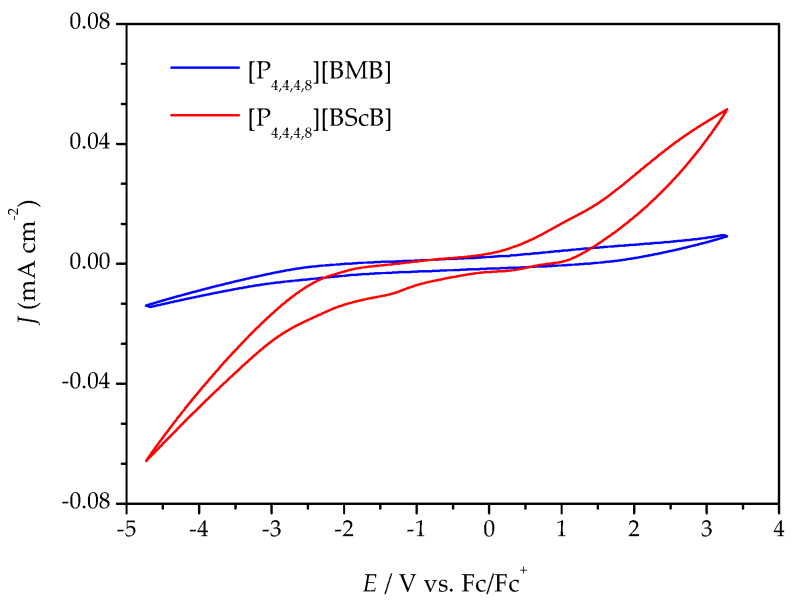
Cyclic voltammograms of the ILs at a GC electrode at 20 °C and a 100 mV s^−1^ potential sweep rate.

Variable temperature cyclic voltammetry (CV) and linear sweep voltammetry (LSV) were both employed to evaluate the electrochemical stabilities of the ILs. Figure 4 illustrates a comparison of the cyclic voltammograms for the [P_4,4,4,8_][BMB] and [P_4,4,4,8_][BScB] ILs at 20 °C (the complete five cycles are shown in Figures S2 and S4). [P_4,4,4,8_][BMB] is clearly electrochemically more stable than [P_4,4,4,8_][BScB] at the surface of the GC electrode. In addition, a significantly higher background current is produced by [P_4,4,4,8_][BScB] than [P_4,4,4,8_][BMB], indicating that the former IL is more electroactive and involved in rapid electron transfer with the electrode surface. The variable temperature cyclic voltammograms for both [P_4,4,4,8_][BMB] and [P_4,4,4,8_][BScB] ILs are shown in Figures S1 and S3, and reveal electrochemical stability and reversibility of the cathodic and anodic reactions occurring in these ILs in the applied potential limits also at elevated temperatures.

**Figure 5:**
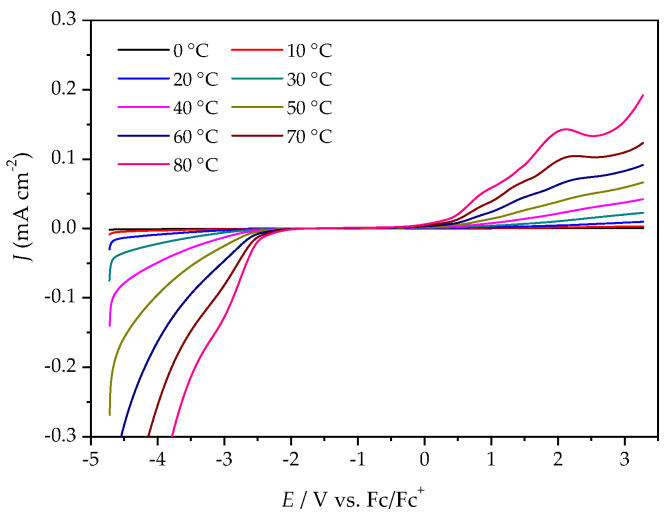
LSV of [P_4,4,4,8_][BMB] at a GC electrode, different temperatures and a scan rate of 20 mV s^−1^.

**Figure 6:**
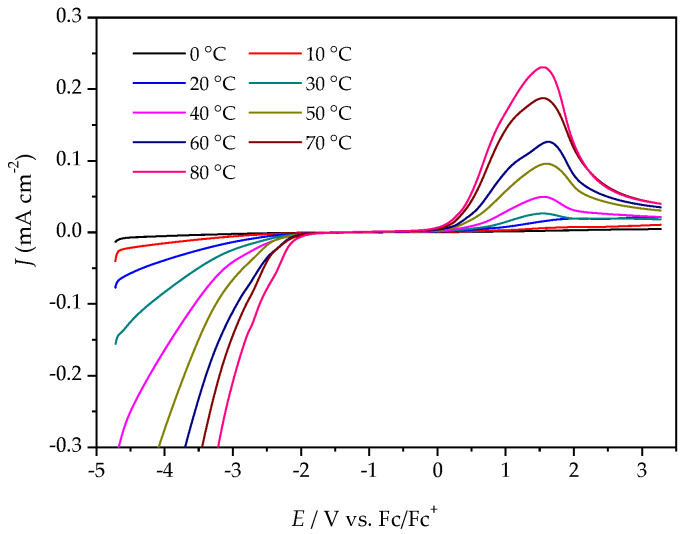
LSV of [P_4,4,4,8_][BScB] at a GC electrode, different temperatures, and a scan rate of 20 mV s^−1^.

The ESWs were determined in more detail by using LSV over a temperature range from 0 °C to 80 °C. The ESW of [P_4,4,4,8_][BMB] is much wider than for [P_4,4,4,8_][BScB] for all temperatures (Figure 5 and Figure 6, Table S1). For example, at 20 °C, the ESWs of [P_4,4,4,8_][BMB] and [P_4,4,4,8_][BScB] are 7.31 V and 3.97 V, respectively. The significantly lower ESW of [P_4,4,4,8_][BScB] is unexpected, as the [BScB]^−^ anion has previously been shown to have excellent chemical stability in comparison to the [BMB]^−^ anion at the surfaces of γ-Al_2_O_3_, MgO and SiO_2_ [27]. Obviously, the applied potential and/or electrode surface makes a difference.

**Figure 7:**
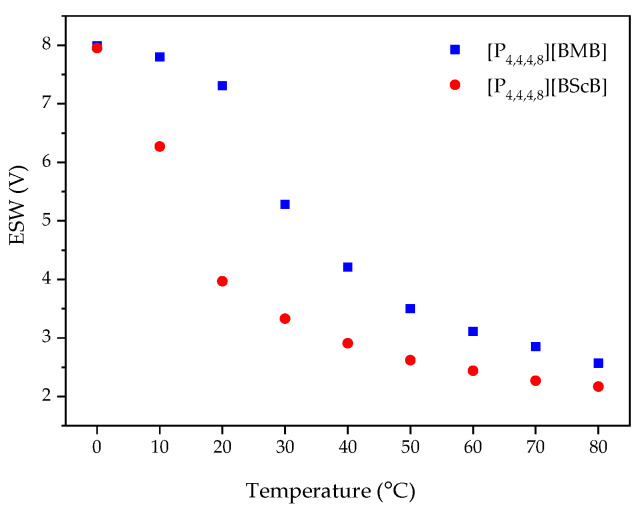
ESWs as a function of temperature for the ILs using a GC electrode.

An increased operating temperature often leads to a narrower ESW, as also predicted by the Nernst equation [39]. For example, Chavan et al. observed a decrease in the ESWs of ILs containing ether and siloxane functionalized imidazolium-based cations and the TFSI anion [40]. Here we observe a sharp decrease in the ESW width for both [P_4,4,4,8_][BMB] and [P_4,4,4,8_][BScB] as a function of temperature (Figure 7), with a large decrease for both ILs between 10 °C and 20 °C. This might be due to the lowered viscosity resulting in a higher ionic conductivity, and the dissociation of ions of the ILs leading to a faster ion diffusivity and more frequent contacts with the surface of electrode. The anodic potential (*E*_A_) is more affected than the cathodic potential (*E*_C_), indicating that the borate anions are more sensitive to the temperature increase than the [P_4,4,4,8_]^+^ cation.

Another plausible reason for the reduction in ESWs at elevated temperatures might be the traces of water present in both these ILs. Schröder et al. found that the ESWs of ILs are significantly decreased in the presence of even very small amount of water [41]. It has also recently been revealed, through systematic molecular dynamic simulations, that the water molecules present in ILs based on [BMB]^−^ and [BScB]^−^ anions are specifically located around the central polar segment of the anions to avoid direct contact with the hydrophobic phenyl rings [42]. Furthermore, the water molecules are embedded in the cavities between ions of the ILs and result in the formation of intermolecular hydrogen bonds between the water molecules and the anions. Such interactions facilitate microscopic liquid structures through ion-water-ion multiple complexes that make it very difficult to remove these water molecules by using vacuum and temperature treatments. Therefore, the shoulder around 1 V in the LSV curves of both [P_4,4,4,8_][BMB] and [P_4,4,4,8_][BScB], more prominent at elevated temperatures, might be due to the release of water molecules at the surface of electrode under the applied potential that previously were strongly trapped in the three-dimensional organization of the ILs [43].

Altogether, the ESW data suggest that [P_4,4,4,8_][BMB] is electrochemically more stable than [P_4,4,4,8_][BScB], while the latter is thermally and chemically more stable. This is in accordance with the results by Mun et al. [44] showing that 1-ethyl-3-methylimidazolium (emim) TFSI has a lower electrochemical stability both at 20 °C and 120 °C as compared to both [C_3_mpyr]TFSI and [C_4_mpip]TFSI, despite that [emim]TFSI is thermally more stable.

## 3. Materials and Methods

### 3.1. Materials

The synthesis and characterization of [P_4,4,4,8_][BMB] and [P_4,4,4,8_][BScB] are described in detail in our previous publication [36]. The samples were kept in a vacuum oven at 80 °C for 5 days prior to measurements. The water contents were 600 ± 50 ppm and 200 ± 40 ppm in [P_4,4,4,8_][BMB] and [P_4,4,4,8_][BScB], respectively, as determined by Karl Fischer titration using 917 coulometer (Metrohm, Herisau, Switzerland). Efforts were made to minimize the amount of water, but due to the strong interactions between the water molecules and the anions of these ILs, it was impossible to remove all traces of water [42].

### 3.2. Thermal Stability

Thermo-gravimetric analysis (TGA) was performed using a Perkin Elmer 8000 TGA (Perkin Elmer, Waltham, MA, United States) apparatus. The dynamic TGA experiments were performed at a heating rate of 10 °C min under nitrogen gas and air. About 4 mg of IL was used for each experiment.

### 3.3. Ionic Conductivity

A Metrohm Autolab PGSTAT302N electrochemical workstation (Metrohm Autolab, Utrecht, The Netherlands) with an FRA32M module for impedance measurements, all controlled by the Nova 2.02 software (Metrohm Autolab, Utrecht, The Netherlands), was used. The ionic conductivities were measured in the frequency range from 1 Hz to 1 MHz using 10 mV_rms_ AC voltage amplitude and in the temperature range from −20 °C to 100 °C. A two-electrode set-up was used with a 2 mm diameter glassy carbon (GC) working electrode and a 70 μL Pt cup as a sample container as well as counter electrode. Both the electrodes were polished with a Kemet diamond paste 0.25 μm prior to each measurement. The cell constant was determined by using a 100 μS cm^−1^ KCl standard solution from Metrohm (Metrohm, Herisau, Switzerland) (Kcell = 1.486 cm^−1^). The cell was thermally equilibrated for 10 min before each measurement.

### 3.4. Electrochemical Stability

A sample of about 70 μL was placed in a sealed TSC 70 cell coupled with temperature-controlled Microcell HC (RHD instruments, Darmstadt, Germany). Cyclic voltammetry (CV) and linear sweep voltammetry (LSV) experiments were performed in the temperature range from 0 °C to 80 °C using a three electrode set-up: GC as a working electrode (WE), Pt cup as a counter electrode (CE) and an Ag wire coated with AgCl was used as a pseudo-reference electrode (RE). The cyclic voltammograms were recorded at a scan rate of 100 mV s^−1^ while the linear sweeps voltammograms were recorded at 20 mV s^−1^. The potential values were converted to ferrocene reference as an internal standard. The ESW limits were determined from LSV curves using a 0.01 mA cm^−1^ cut-off current density [45].

## 4. Conclusions

Two phosphonium-orthoborate ILs, [P_4,4,4,8_][BMB] and [P_4,4,4,8_][BScB], with the same cation and structurally very similar anions, show different thermal and electrochemical stabilities. [P_4,4,4,8_][BScB] has a higher thermal stability both in nitrogen atmosphere and in air, and higher ionic conductivity, but has lower electrochemically stability. On the other hand, [P_4,4,4,8_][BMB] exhibits higher electrochemical stability, but lower thermal stability and ionic conductivity. As expected, the ESW of both ILs significantly decrease as a function of temperature. These findings suggest that there is no direct relation between thermal stability, chemical reactivity, and electrochemical stability. An IL can be thermally less stable, but chemically more stable, and vice versa. This study suggests that [P_4,4,4,8_][BMB] is suitable for applications where high electrochemical stability is required and [P_4,4,4,8_][BScB] can be used in applications where large electrochemical stability is not very important, but where thermal and chemical stability is essential.

## Figures and Tables

**Table 1:** VFT equation parameters and activation energies for the ionic conductivity data of the ILs.

IL	σ_0_ (S cm^−1^)	*B* (K)	*T*_0_ (K)	*E*σ (kJ mol^−1^ K^−1^)
[P_4,4,4,8_][BMB]	1.15	1460	189	12.1
[P_4,4,4,8_][BScB]	0.50	1290	182	10.7

## Supplementary Materials

The following are available online at https://www.mdpi.com/1420-3049/25/10/2388/s1.
