# Supplementary material for: Comparing the Thermal and Electrochemical Stabilities of Two Structurally Similar Ionic Liquids
Source: Molecules. 2020 May 21;25(10):2388. doi: 10.3390/molecules25102388 (PMC7287963; doi:10.3390/molecules25102388)
Supplement: Supplementary file 1 [file molecules-25-02388-s001.pdf]

Supplementary Material

Comparing the Thermal and Electrochemical Stabilities of Two Structurally Similar Ionic Liquids

Faiz Ullah Shah[1,*], Inayat Ali Khan[1] and Patrik Johansson[2,*]

[1] Chemistry of Interfaces, Luleå University of Technology, SE-971 87 Luleå, Sweden

[2] Department of Physics, Chalmers University of Technology, SE-412
96 Gothenburg, Sweden

*Correspondence: faiz.ullah@ltu.se (F.U.S); patrik.johansson@chalmers.se (P.J)

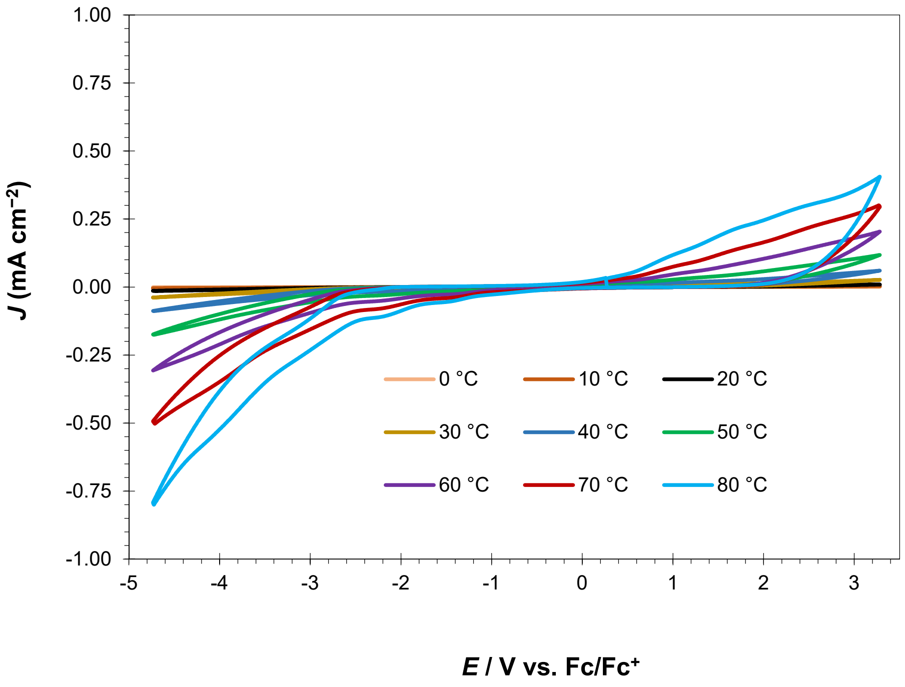

Figure S1. Variable temperature CVs of [$P_{4,4,4,8}$][BMB] on GC at 100 mV s^{-1} scan rate.

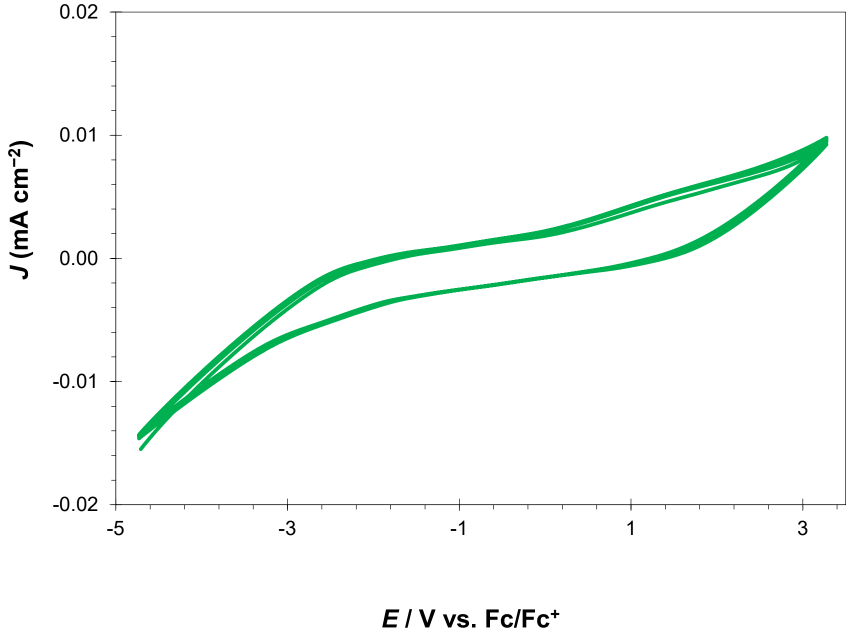

Figure S2. CVs (5 cycles) of [$P_{4,4,4,8}$][BMB] on GC at 100 mV s^{-1} scan rate and 20 °C.

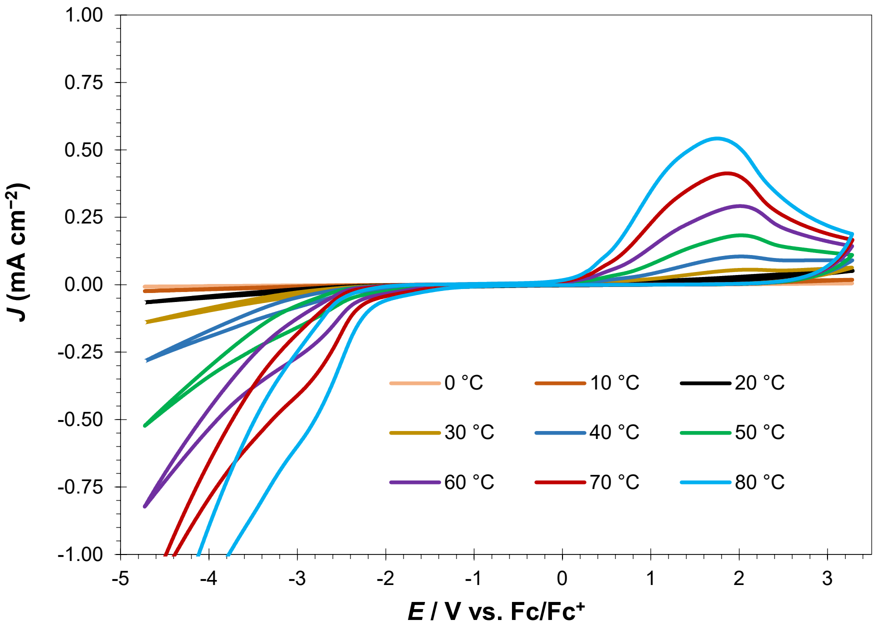

Figure S3. Variable temperature CVs of [$P_{4,4,4,8}$][BScB] on GC at 100 mV s^{-1} scan rate.

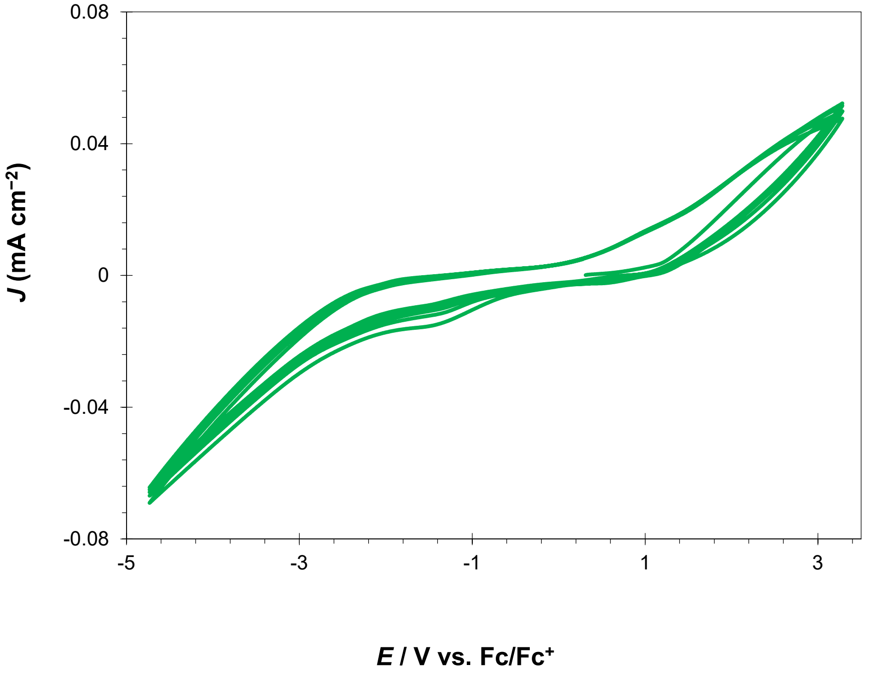

Figure S4. CVs (5 cycles) of [$P_{4,4,4,8}$][BScB] on GC at 100 mV s^{-1} scan rate and 20 °C.

Table S1. Electrochemical stability limits and ESWs of the ILs at different temperatures.

Temperature (°C)	$[P_{4,4,4,8}][BMB]$			$[P_{4,4,4,8}][BScB]$		
	E_A (V)	E_C (V)	ESW (V)	E_A (V)	E_C (V)	ESW (V)
0	3.27	−4.72	7.99	3.27	−4.68	7.95
10	3.25	−4.55	7.80	3.12	−3.51	6.27
20	3.13	−4.18	7.31	1.17	−2.82	3.97
30	1.95	−3.33	5.28	0.83	−2.50	3.33
40	1.21	−3.00	4.21	0.55	−2.36	2.91
50	0.80	−2.70	3.50	0.27	−2.35	2.62
60	0.56	−2.55	3.11	0.26	−2.18	2.44
70	0.40	−2.45	2.85	0.10	−2.17	2.27
80	0.21	−2.36	2.57	0.09	−2.08	2.17
